# Coloring coral larvae allows tracking of local dispersal and settlement

**DOI:** 10.1371/journal.pbio.3001907

**Published:** 2022-12-06

**Authors:** Christopher Doropoulos, George Roff

**Affiliations:** CSIRO Oceans & Atmosphere, St Lucia, Australia; University of Washington Seattle Campus: University of Washington, UNITED STATES

## Abstract

Quantifying patterns of dispersal and settlement in marine benthic invertebrates is challenging, largely due the complexity of life history traits, small sizes of larvae (<1 mm), and potential for large-scale dispersal (>100 km) in the marine environment. Here, we develop a novel method that allows for immediate differentiation and visual tracking of large numbers of coral larvae (10^6^ to 10^9^) from dispersal to settlement. Neutral red and Nile blue stains were extremely effective in coloring larvae, with minimal impacts on survival and settlement following optimization of incubation times and stain concentrations. Field validation to wild-captured larvae from the Great Barrier Reef demonstrates the efficacy of staining across diverse taxa. The method provides a simple, rapid (<60 minutes), low-cost (approximately USD$1 per 10^5^ larva) tool to color coral larvae that facilitates a wide range of de novo laboratory and field studies of larval behavior and ecology with potential applications for conservation planning and understanding patterns of connectivity.

## Abstract 2

This study describes develop a novel method to color large numbers of coral larvae (millions to billions), allowing immediate differentiation and visual tracking of coral larvae from dispersal to settlement, with the potential to facilitate a wide range of de novo studies of larval behavior and ecology.

## Main

Increases in the frequency and intensity of anthropogenic disturbances over the past century has led to widespread fragmentation and habitat loss throughout the world’s oceans [1,2]. In marine ecosystems, the persistence and recovery of populations following disturbance is strongly dependent on the dispersal of propagules from adjacent habitats [3,4]. As such, quantifying the spatial patterns of connectivity among habitats is of key importance for management planning and conservation of marine ecosystems [3,5,6]. To date, a range of indirect methods including simulation modelling [6], elemental fingerprinting [4], and population genetic approaches [7] have been used to infer patterns of connectivity across a range of temporal and spatial scales. However, for many important habitat-forming benthic marine invertebrates such as corals, sponges, and bivalves, direct quantification of larval dispersal has remained a major challenge [5]. Benthic marine invertebrates have complex bipartite life histories [4] with high reproductive output (>10^6^ per individual), small sizes (typically <1 mm), and low survival (<1%) [3,5], requiring the tracking of millions of larvae for direct quantification of dispersal. This challenge is further complicated by complex oceanographic currents [6] and extended pelagic duration phases of upwards of 100 days [8], resulting in dispersal ranging from 10^−1^ to 10^3^ km [9].

The severe impacts from climate change to coral reefs worldwide in the 21st century has resulted in major population loss and reduced recovery potential [2]. Consequently, research focuses have shifted towards large-scale restoration efforts that can mitigate against future disturbances and repopulate damaged reefs [10–14]. Of these interventions, larval reseeding is among the most promising large-scale approaches to regenerate depleted coral populations and reestablish breeding populations [10,15–17], largely due to exceptionally high reproductive output of corals (as high as 10^6^ to 10^7^ eggs per colony; [18]). This approach captures larvae from wild slicks or from gametes released from gravid colonies in aquaria, cultures the larvae until they are competent, and then those competent larvae are released onto reefs to settle over a period of 24 to 120 hours [10,15–17,19]. Despite the potential for larval reseeding in large-scale restoration, quantifying the impact of the approach at local to regional scales is complex as reseeded larvae are indistinguishable from natural background larval settlement.

To overcome these limitations, we developed and validated a novel method for coloring coral larvae that allows for differentiating among sources and species. Through differential vital staining of coral larvae, our method allows for direct tracking of local dispersal through to settlement of larvae onto coral reef substrates. Additionally, the method directly facilitates visual differentiation among larval cohorts and between coral species by assigning multiple colors, facilitating parentage assignment and allowing for de novo studies of behavior and ecological interactions in both laboratory and field studies. We initially tested four vital stains (neutral red, Nile blue, calcein blue, and alizarin red), followed by a series of laboratory experiments to optimize the visual efficacy of the two most successful stains (neutral red and Nile blue) across a range of stain concentrations and larval incubation times that minimized any adverse impacts on larval survival and settlement. Our method was then validated in the field by coloring wild-caught larvae from natural coral spawn slicks and tracking colored larvae to settlement on reef substrates. The method provides a rapid, simple, nontoxic, and low-cost (approximately USD$1 per 10^5^ larva) approach with potential to differentiate cohorts and directly quantify fine-scale dispersal in large numbers (10^6^ to 10^8^) of coral larvae.

## Results

### Initial staining procedures

To establish the potential for staining of coral larvae, an initial factorial experiment was conducted using four stains (neutral red, Nile blue, Alizarin red, and calcein blue). By varying stain concentrations (1, 10, 100 mg l^−1^) and incubation times (1, 6, 12, 24 hours) while quantifying larval survival and settlement, we aimed to test the effects of visual staining while reducing the potential for immediate and latent toxic effects on coral larvae. As corals exhibit high patterns of diversity, our initial experiments included two common yet phylogenetically [20] and functionally [21] distinct species of coral: *Acropora spathulata* (family: Acroporidae, corymbose growth form) and *Platygyra daedalea* (family: Merulinidae, massive growth form). Both species were collected at Heron Island (southern Great Barrier Reef) and larval staining was conducted 5 days after spawning once the larvae had developed their sensory and motility systems.

**Fig 1:**
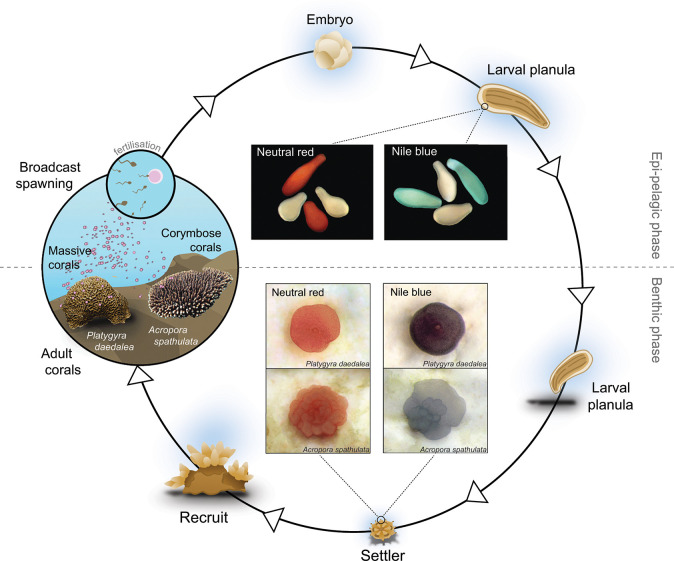
Life history strategy of broadcast spawning corals, from the epipelagic release phase of sperm and eggs in mass spawning to the benthic stage of larval settlement and recruitment into the population. Inset photographs are representative images of neutral red and Nile blue stains on larvae (colored vs. control unstained larvae in white) and recently settled life history phases. Clip art has been modified from [22] under a Creative Commons Attribution (CC BY) license. Images (adult, larvae, settler) supplied by authors.

**Fig 2:**
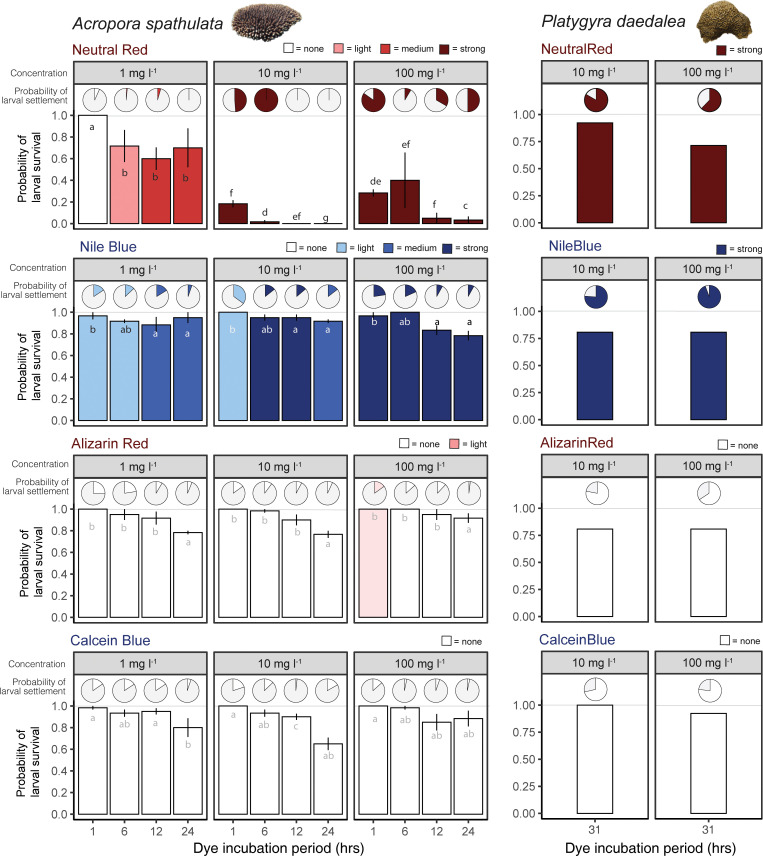
Probability of larval survival and settlement for two species of coral exposed to four stains at different concentration levels and incubation times. For probability of settlement, the filled proportion of the circle indicates the proportion of surviving larvae, and colors indicate the strength of the larval stain at each stage (larval stage and settled larvae; inset key indicates none, light, medium, or strong staining). Letters in bars indicate pairwise differences in probability of larval survival for *A*. *spathulata*. When a *p* value exceeds α = 0.05, then two means have at least one letter in common. Images supplied by authors. Data underlying this Figure can be found at https://doi.org/10.25919/4rry-xg84.

Larval staining of *A*. *spathulata* and *P*. *daedalea* was highly effective using neutral red and Nile blue, with larvae and newly settled corals easily distinguishable compared to their natural counterparts (Fig 1). Survival of *A*. *spathulata* larvae was reduced at longer incubation times and higher concentrations of dyes (χ^2^ = 238.75, df = 18, *p* < 0.001). The visual effect of the neutral red was most obvious at higher concentrations (10 and 100 mg l^−1^) yet led to significantly reduced larval survival even at short incubation periods (Figs 2 and A in S1 Text). At the lowest concentration (1 mg l^−1^), neutral red achieved light staining after 6 hours, and medium levels of staining after 12 hours of incubation with >60% larval survival. Nile blue achieved the most consistent staining effect of all stains, with light stains observed at the lowest concentration (1 mg l^−1^) after a single hour of incubation (Fig 2). Survival was high in Nile blue across all concentrations (Fig 2) and did not differ from controls (unstained larvae) after 12 hours (Fig B in S1 Text). Alizarin red and calcein blue failed to have any measurable visual effects on larvae regardless of concentration and incubation time (Fig A in S1 Text). Larval survival did not differ from controls at the 12-hour time point (Fig B in S1 Text), and survival decreased with increasing incubation time in treatments (Fig 2).

Larval settlement for *A*. *spathulata* differed among treatments (χ^2^ = 227.49, df = 18, *p* < 0.001) and was consistently low (Fig 2), with most treatments resulting in <20% settlement and more than half exhibiting less than 10% settlement (Figs 2 and A in S1 Text). No significant differences were observed in the probability of settlement between control and stained larvae with the exception of neutral red, where settlement was significantly lower than the controls due to lower initial survival rates (Fig B in S1 Text).

In contrast to *A*. *spathulata*, larvae of *P*. *daedalea* were considerably more robust, with >75% survival (Fig 2) even at longer incubation times (31 hours versus 24 hours; Fig 2). Similar to *A*. *spathulata*, neutral red and Nile blue resulted in strong staining at intermediate and high concentrations (10 and 100 mg l^−1^; Fig 2), while Alizarin red and calcein blue failed to color larvae. In contrast to *A*. *spathulata*, larval settlement of *P*. *daedalea* was considerably higher, with >70% settlement rates across all stains and concentrations (Fig 2). Trials of mixed red and blue *P*. *daedalea* larvae placed with preconditioned settlement tiles showed that larval sources can be readily distinguished by utilizing their coloration (Fig C in S1 Text).

### Refined staining procedures

Following the initial success of neutral red and Nile blue stains, we conducted a second experiment to refine the staining procedure to include a wider range of corals: *Acropora anthocercis*, *Coelastrea aspera*, *Dipsastraea favus*, and *Platygyra sinensis*, collected from the central Great Barrier Reef. These taxa are functionally distinct [21] (tabular growth form: *A*. *anthocercis*, massive growth forms: *C*. *aspera*, *D*. *favus*, *P*. *sinensis*) and phylogenetically distant [20] (family: Acroporidae and family: Merulinidae). Based on the outcomes of the first experiment, we reduced the incubation times to 5 to 30 minutes and concentrations to 1 to 100 mg l^−1^ for larvae stained with neutral red, and 60 to 120 minutes and 1 to 1,000 mg l^−1^ for larvae stained with Nile blue.

**Fig 3:**
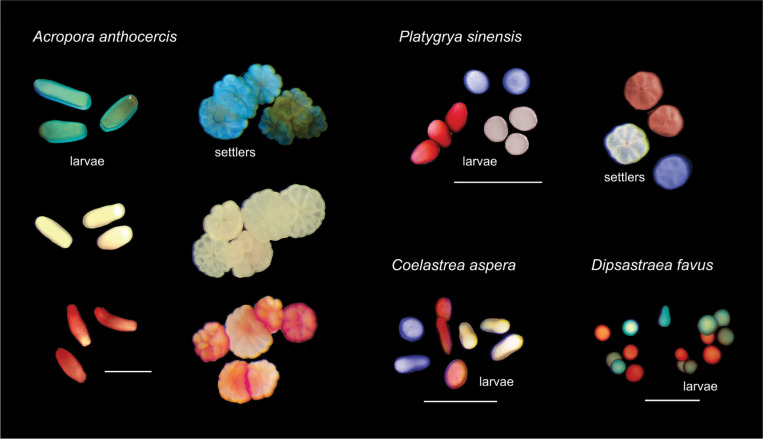
Representative images of free-swimming and newly metamorphosed larvae (Nile blue, unstained, neutral red) from *A*. *anthocercis*, *P*. *sinensis*, *C*. *aspera*, and mixed Nile blue and neutral red stained *D*. *favus* larvae. White scale bars = 1 mm. Images supplied by authors.

**Fig 4:**
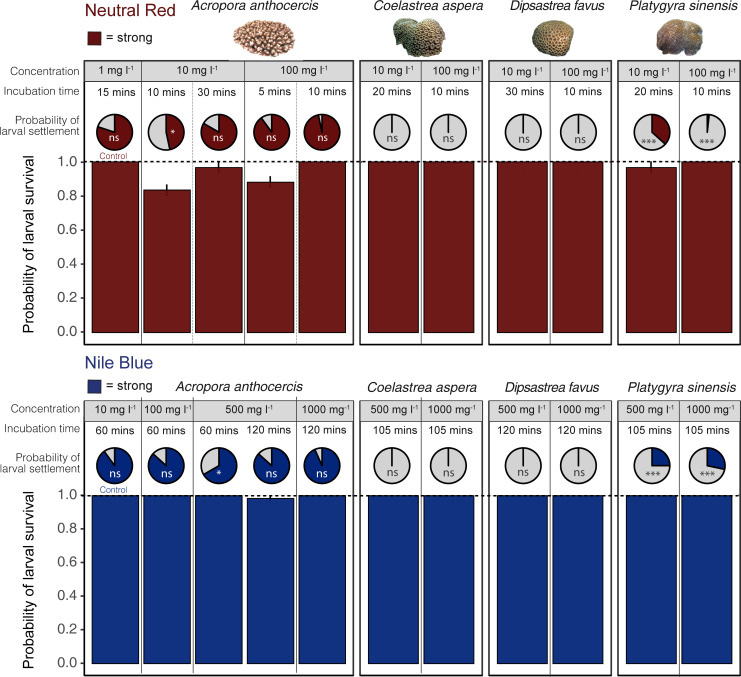
Probability of larval survival and settlement for four species of coral exposed to neutral red and Nile blue stains at different concentration levels and incubation times. For probability of settlement, the filled proportion of the circle indicates the proportion of surviving larvae. Differences in larval survival between treatments shown against controls (dash line), where no mortality was observed (100% survival). Inset notation for probability of settlement indicates significant differences from control within each species at an α of 0.05 (^ns^ = no significant difference, * = *p* < 0.05, ** *p* < = 0.01, *** = *p* <0.001). Images supplied by authors. Data underlying this Figure can be found at https://doi.org/10.25919/4rry-xg84.

Under the refined staining procedures, the visual effect of larval staining was optimized across the diverse range of taxa (Fig 3). Survival was consistently high (>80%) with no difference among species and treatments (Fig 4), whereas larval settlement varied among species and treatments (χ^2^ = 46.2, df = 6, *p* < 0.0001; Fig D in S1 Text). For *A*. *anthocercis*, larval settlement was high (>75%) across all treatments and did not differ from controls with the exception of a single treatment (Fig 4). Larval settlement of *P*. *sinensis* in the neutral red stain was significantly higher in one treatment (10 mg l^−1^ for 20 minutes), yet significantly lower in the other treatment (100 mg l^−1^ for 10 minutes), indicating that higher stain concentrations may reduce larval settlement. Larval settlement of *P*. *sinensis* in the Nile blue stain was significantly higher in both treatments than in the control (Fig 4). Both stained or unstained (control) larvae of *C*. *aspera* and *D*. *favus* failed to settle for the duration of the experiment, suggesting the larvae were not competent and/or unresponsive to the crustose coralline algae cue.

### Field validation with wild coral larvae

**Fig 5:**
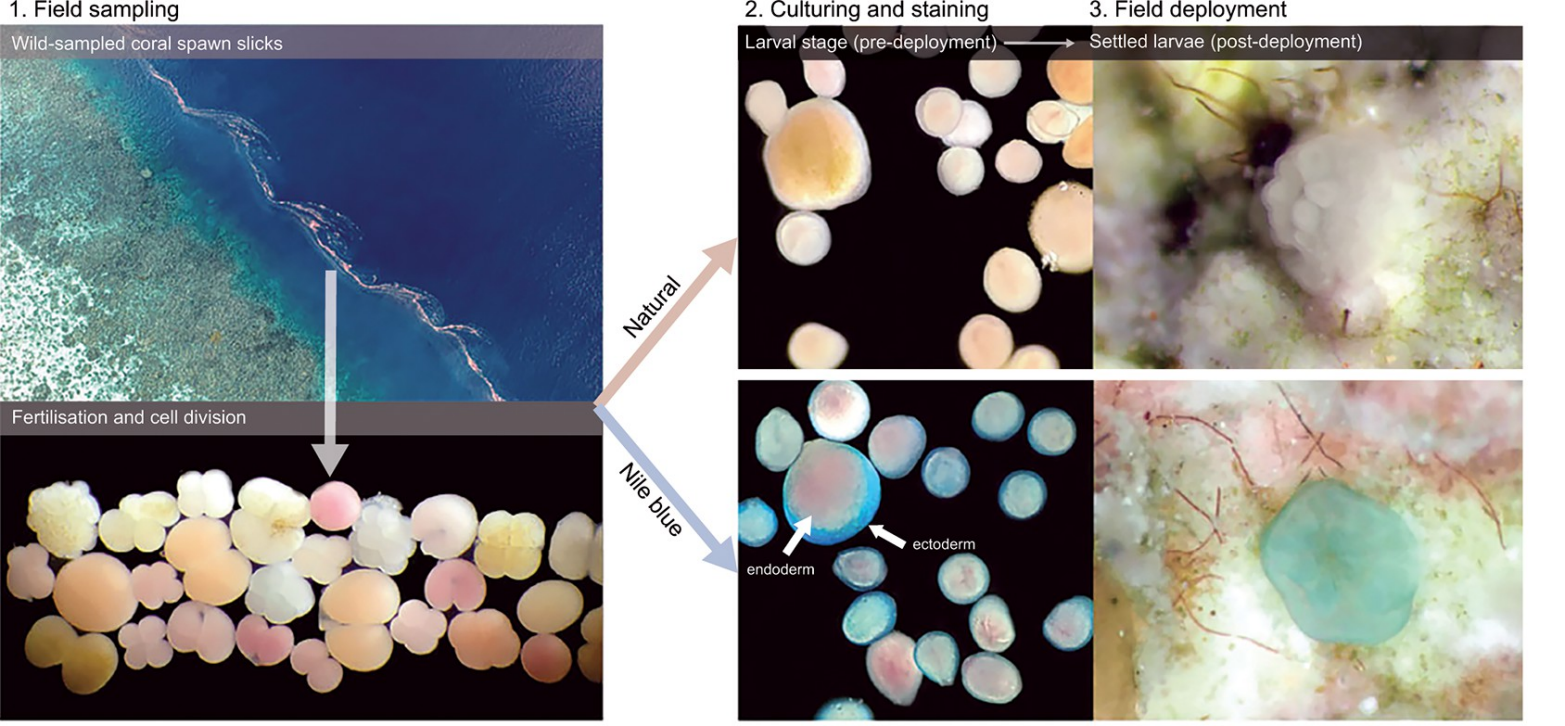
Field validation of larval staining: capture of wild-sampled coral spawn slicks showing high diversity of developing coral embryos, lab-based staining using Nile blue (1,000 mg l^−1^ concentration for 60 minutes), and field deployment of competent stained and natural (unstained) larvae for detection on reef substrates. Images supplied by authors.

While our lab-based experiments on small numbers (*n* < 100) of cultured larvae clearly reveal the potential of coloring larvae during dispersal and settlement, the applicability of the staining procedure for tracking broadscale dispersal of large numbers (*n* > 10,000) of larvae in natural environments required (i) validation of coloration against natural diverse coral larvae collected from wild spawn-slicks and (ii) a field validation of larval settlement within a natural coral reef environment (Fig 5). Developing larvae were collected from wild coral spawn slicks adjacent to Lizard Island (northern Great Barrier Reef) and cultured in larval pools on the reef. After 6 days larval development, approximately 10,000 larvae were subsampled from the culture pool (estimated 1.5 million total) and stained with Nile blue (1,000 mg l^−1^ for 60 minutes) to contrast against the natural colors of coral larvae.

Visual assessment of wild coral larvae under light microscopy confirmed the presence of a diverse multispecies larval assemblage (size range: 150 to 650 μm), ranging from cream to pink to red in coloration. Nile blue staining of these wild-captured larvae was highly effective, with >98% of larvae showing discernible staining effects (Fig 5). Following larval release onto a lagoonal patch reef, settlement of stained larvae was detected on settlement tiles to validate the application. While the common, smaller cream-colored larval species were effectively stained entirely blue in color, the larger less common “red” larval species appear to be stained blue in the outer ectodermal layers only, with the underlying, red-pigmented endodermal layers remaining partially visible (Fig 5).

## Discussion

We outline an optimized methodology that allows for differentiating larval cohorts and direct tracking of dispersal and settlement of broadcast spawning corals. Importantly, the method is validated in both controlled laboratory experiments and in a natural field environment across a range of functionally [21] distinct and phylogenetic distant lineages [20] of corals. To our knowledge, this study represents the first direct tracking of coral larvae from pelagic stage to benthic settlement on a coral reef, facilitating a range of de novo studies from elucidating small-scale patterns of larval settlement at the scale of millimeters to tracking dispersal at the scale of meters to kilometers.

To be effective in differentiating among larval cohorts, the proposed method should be (i) direct and easily detectable and (ii) low in toxicity. Coloring coral larvae with vital stains allows for rapid and simple visual differentiation among larval cohorts, with Nile blue and neutral red stained larvae clearly visible from natural larvae with the naked eye (Figs 1, 3, 5, A, and C in S1 Text) despite the small size of coral larvae (300 to 900 μm; [22]). The peak emission spectra of neutral red (610 to 630 nm; [23]) and Nile blue (650 to 670 nm; [24]) are distinct from the spectral signatures of green fluorescent proteins in *Acropora millepora* larvae (510 to 520 nm; [25]), highlighting their potential use as fluorochromes in cellular imaging or cytometry applications. For example, combing our method with large-particle flow cytometry of live larvae [26] would enable sorting of larval cohorts in experiments to assign parentage, or allow rapid separation to recover experimentally colored larvae from mixed wild cultures in large-scale field experiments. Following refinement of concentration and exposure times, our results indicate that for two vital stains (neutral red and Nile blue), coloring coral larvae has minimal direct (reduced larval survival) or indirect (latent effects on settlement and metamorphosis) impacts, with no clear differences observed from controls. At higher concentrations and incubation times, toxicity differed among coral taxa: larvae from the family Acroporidae exhibited greater sensitivity to neutral red than Merulinidae. The toxicity of neutral red to Acroporidae larvae is counter to that reported from other benthic marine invertebrate larvae (e.g., oysters) that exhibit greater sensitivity to Nile blue [27] and are unaffected by neutral red [28]. While calcein blue and alizarin red have been successfully used in staining adult benthic invertebrates [27–29], as well as mineral deposits found within brooded coral planulae [30], the absence of visual staining in spawning coral larvae due to a lack of calcium binding potential limits their application to post-settlement life history stages (Fig 1) following the onset of early skeletal formation [31].

To be effective in directly tracking larval dispersal, the proposed method must be (i) procedurally simple and rapid; (ii) easily detectable in field settings; (iii) easily scalable to large numbers (10^6^ to 10^9^) of larvae; (iv) nontoxic in the marine environment; (v) widely available; and (v) cost effective. From a procedural perspective, the protocol is simple and rapid (<60 minutes incubation), allowing for application in remote field locations where laboratory facilities are unavailable. In terms of detection, larvae can be detected following release by sampling with plankton tows during the pelagic stage [32] and directly on reef substrates post-settlement using settlement tiles [33] or relatively inexpensive (<USD$500) underwater cameras (e.g., Fig 5). From a scalability perspective, staining of larvae at relatively low concentrations (<1,000 mg l^−1^) allows for bulk staining of large numbers of larvae (e.g., 10^5^ larvae per 20 l container) for use in large-scale deployments in local dispersal tracking and restoration efforts [10]. From a toxicity perspective, at the low concentrations and/or short incubation times, the vital stains are nontoxic and allow for use and dispersal in the marine environment. From the perspective of cost and availability, both vital stains are widely available and easily transported in powder form, and highly cost effective for large deployments costing approximately USD$1 to stain 10^5^ larvae. The simplicity and low cost associated with staining and detection will allow enable uptake of the method in developing coral reef nations that often require low-cost and low-technology approaches (e.g., [16,34]).

While the method is widely applicable to a range of ecological, behavioral, and physiological studies, the application of colored larvae to applied restoration activities requires further assessment alongside large-scale field deployments. First, as coral eggs and larvae are a source of food for planktivorous reef fish during coral spawning [35], coloring larvae may enhance predation compared with natural colored larvae, potentially depleting larval pools and negatively impacting density-dependent settlement processes. Similarly, newly settled corals that are colored may also be more easily detectable by invertebrate [36] and fish [37] predators. Secondly, while our optimization process across the laboratory experiments demonstrates that there are no latent effects of coloring the larvae on settlement, there may be longer-term latent effects on post-settlement growth and survival. Finally, while our results here demonstrate the efficacy of the approach over short time periods (5 days) following metamorphosis and settlement, the longer-term effectiveness and retention of coloration across weeks to months is unknown. Earlier studies applying neutral red and Nile blue on oysters and starfish indicate retention of larval stains can last up to 70 days with larvae and 6 months following metamorphosis [28]. However, the uptake of intracellular algae (Symbiodinium) following settlement and high rates of cell division accompanying rapid initial growth and division of newly settled coral polyps may limit the potential of vital staining beyond the first 30 days of settlement. The degree to which these points will influence large-scale deployments will be context dependent and likely vary among coral species: for example, the impact of staining on predation on newly settled larvae may have more impact on species with less cryptic settlement preferences, while the detectability of coloration over time may diminish more rapidly in faster growing species. Deployment of colored larvae in field settings will require adequate controls and careful consideration of these latent ecological effects.

While the method outlined here has clear application to studies of larval dispersal and larval restoration, the method has potential for a wide range of de novo insights into larval ecology and behavior. The ability to use different color stains to differentiate between larval cohorts will enable fine-scale examination of density-dependence and conspecific interactions during settlement [8], while coloring phylogenetically similar species will provide novel insights into intraspecific competition and facilitation, and elucidating spatial patterns of settlement on natural reef substrates. As additional experiments investigate knowledge gaps around potential predator and latent effects of the staining approach on larvae, at larger scales, the method should be applicable as a low cost applied approach to trial direct validation of modelling studies of fine-scale connectivity [6,32,38,39] and quantifying the success of larval restoration methods by allowing for immediate differentiation between targeted larval releases and background settlement. As increases in marine heatwaves under future climate change will result in populations becoming ever more spatially fragmented, the need to quantify dispersal and connectivity with a view to optimizing management strategies for conservation planning of coral reefs will become increasingly important.

## Methods

### Vital stains

The following vital stains and protocols were selected for experimentation with coral larvae: Nile blue [40] (C_20_H_20_ClN_3_O, CAS number: 3625-57-8), neutral red [41] (C_15_H_17_N_4_, CAS number: 553-24-2), alizarin red [27] (C_14_H_8_O_4_, CAS number: 72-48-0), and calcein blue [29] (C_15_H_15_NO_7_, CAS number: 54375-47-2).

### Initial staining experiment

To establish the potential for larval staining, an initial factorial experiment was conducted using four stains at differing concentrations and incubation times. These experiments were conducted on two common species of coral, *A*. *spathulata* (family: Acroporidae, corymbose growth form) and coral *P*. *daedalea* (family: Merulinidae, massive growth form). Both species were collected from the reef flat at Heron Island (southern Great Barrier Reef under permit number G19/42916.1) and maintained in 50 l aquaria prior to spawning. *A*. *spathulata* spawned on 17 November 2019 (21:45 to 00:40), and *P*. *daedalea* spawned on 19 November 2019 (18:30 to 18:40). In both species, larval staining began 5 days after spawning once larvae were developed.

*A*. *spathulata* larvae were placed with 10 ml of solution in individual scintillation vials for 1-, 6-, 12-, and 24-hour incubation periods at three different stain concentrations (1, 10, and 100 mg l^−1^), a total of 12 treatments for each vital stain. Three replicates were conducted per treatment (incubation time * stain concentration), with 20 larvae assigned to each replicate (36 treatments, 720 larvae total). Across all stains, this resulted in 144 treatments, totaling 2,880 larvae. Staining was conducted in independent glass scintillation vials (10 ml total volume). Larvae were added to each treatment so that the end point of the staining was the same across all treatments—i.e., larvae were added to the 1-hour treatment at hour 23, 6-hour treatment at hour 18, and 12-hour treatment at hour 12, at which point larvae were 6 days old. The intensity of staining for each replicate was scored ordinally by a single observer (CD) into four categories: (1) no stain; (2) light staining; (3) medium staining; and (4) strong staining. To quantify the effects of the stain on larval survival, a control with 20 unstained *A*. *spathulata* larvae (*n =* 3 replicates) was conducted at the 12-hour time point. The proportion of alive larvae was counted under a dissecting microscope to determine the effects of staining on larval survival. To determine the effects of stain treatments on larval settlement, surviving larvae from each treatment were then added to individual containers in 250 ml of filtered (0.20 μm) seawater, each with a settlement tile that had been conditioned for 2 months at 5 m depth. Water changes were conducted after 2 days (8 days after spawning), and larval settlement was scored 3 days after tiles were introduced (9 days after spawning).

*P*. *daedalea* larvae were placed with 20 ml stain in wells of cell culture plates (Fig D in S1 Text) for a single 36-hour incubation period at two different stain concentrations (10 and 100 mg l^−1^). For each of the four stains, a single replicate was conducted per each treatment (*n* = 150 larvae per each treatment, 4 treatments, 480 larvae total). Across all stains, this resulted in 16 treatments and 1,920 larvae total. Larval staining, scoring of stained larvae, and larval settlement followed the same protocol as for the *A*. *spathulata* experiment, with the exception of settlement, where larvae were settled on small chips (0.25 cm^2^) of *Porolithon onkodes* crustose coralline algae in sterile 20 ml cell culture wells.

To quantify significant differences between survival and settlement within each stain across different concentrations and incubation times for *A*. *spathulata*, we used a binomial generalized linear model (GLM) for each stain, where incubation time and stain concentration were considered as fixed effects. Models were fit in R (v4.1.2) using the “glm” function in the stats package [42], and Tukey post hoc pairwise differences between treatments (incubation times and concentrations) were tested using the “glht” function and visualized using the “cld” functions in the multcomp package [43].

### Refining staining experiment

To further refine the staining method, we conducted a follow-up experiment in November 2021 at the SeaSim aquaria facility (Australian Institute of Marine Science, Townsville, Australia). Based on the results of the initial experiments, two stains were discarded (alizarin red and calcein blue) and two were selected for further refining of incubation time and stain concentration (neutral red and Nile blue). To explore taxonomic differences in staining potential, four different coral species were used in the second experiment: *A*. *anthocercis* (spawning time: 22:30, 20 October 2021), *D*. *favus* (spawning time: 20:00, 23 October 2021), *C*. *aspera* (spawning time: 21:30, 24 October 2021), and *P*. *sinensis* (spawning time: 22:00, 24 October 2021). These taxa are functionally distinct (tabular growth form: *A*. *anthocercis*; massive growth forms: *D*. *favus*, *P*. *sinensis*, and *C*. *aspera)* and are phylogenetically distant (family: Acroporidae and family: Merulinidae). A table of the following stain times × concentrations for each taxa below is found in Table A in S1 Text. *A*. *anthocercis* neutral red staining was conducted at the following concentrations and incubation time treatments: 1 mg l^−1^ for 15 minutes, 10 mg l^−1^ for 10 and 30 minutes, and 100 mg l^−1^ for 5 and 10 minutes, and a control (6 treatments, 180 larvae total). Nile blue staining was conducted at the following concentrations and incubation time treatments: 10, 100, and 500 mg l^−1^ for 60 minutes, and for 500 mg and 1,000 mg l^−1^ for 120 minutes (5 treatments, 150 larvae total).

*C*. *aspera* neutral red staining was conducted at 10 mg l^−1^ for 20 minutes, and 100 mg l^−1^ for 10 minutes, and a control (3 treatments, 180 larvae total). Nile blue staining was conducted at 500 mg and 1,000 mg l^−1^ for 105 minutes (2 treatments, 120 larvae total). *D*. *favus* neutral red staining was conducted at 10 mg l^−1^ for 30 minutes, and 100 mg l^−1^ for 10 minutes, and a control (3 treatments, 180 larvae total). Nile blue staining was conducted at 500 mg l^−1^ and 1,000 mg l^−1^ for 120 minutes (2 treatments, 120 larvae total). *P*. *sinensis* neutral red staining was conducted at 10 mg l^−1^ for 20 minutes, and 100 mg l^−1^ for 10 minutes (3 treatments, 180 larvae total). Nile blue staining was conducted at 500 mg l^−1^ and 1,000 mg l^−1^ for 105 minutes (2 treatments, 120 larvae total). Across all species, this resulted in 22 treatments and 1,260 total larvae.

In each treatment, larvae were placed in 15 ml of stain solution in individual 6-well culture plates (Fig E in S1 Text). To quantify the effects of the stain on larval survival, controls (*n =* 3 replicates) with unstained larvae (*n* = 10 for *A*. *anthocersis*, *n* = 20 larvae for other taxa) were conducted alongside staining experiments. Larval staining, scoring of stained larvae, and larval settlement followed the same protocol as for the first experiment, with small chips of *P*. *onkodes* (0.25 cm^2^) used to induce settlement. The intensity of stain for each replicate was scored ordinally by a single observer (CD), following the same protocol as the first experiment, and imaged using Toupview software (ToupTek Photonics, Zhejiang, China) on a dissecting microscope. White balance and black balance were set within the software prior to imaging. To test for differences in larval survival and larval settlement among treatments, we used a binomial GLM using the “glm” function in the stats package in R (version 4.1.2). Post hoc differences between treatments (differing incubation times and concentrations) and controls were tested using the “glht” function and visualized using the “cld” function in the emmeans package.

### Wild larval staining experiment

To quantify the efficacy of larval staining on natural wild collected larvae, we sampled multispecies coral slicks from the lagoon at Lizard Island (northern Great Barrier Reef, 14° 41.045′ S, 145° 27.843′ E) on the fourth night after full moon (23 November 2021). Eggs were passively collected in the evening using a passive boom system and cultured in situ in the Lizard Island lagoon in a larval culture pool (5 × 5 m). After 6 days, approximately 10,000 larvae were subsampled from the culture pool (estimated 1.5 million total) for larval staining. Larvae were stained with Nile blue (Fig E in S1 Text) using a concentration of 1,000 mg l^−1^ for 60 minutes to contrast the natural larval colors. Stained and natural larvae were visually assessed under light microscopy. Stained larvae were then released within a net enclosure (2 × 2 m, 125 μm plankton mesh size) containing preconditioned (2 months) settlement tiles (5 × 5 cm) within Lizard Island lagoon to retain larvae during the transition from planktonic larvae to benthic settlement. The net was removed 48 hours following deployment and benthic substrates, and tiles were imaged in situ using an Olympus TG-6 underwater camera in “Microscope Control Mode” (maximum ×28 magnification) to detect the presence of newly settled larvae. Preconditioned tiles were transferred to the lab and assessed under light microscopy for settlement of colored larvae.

## Supporting information

S1 Text Supplementary tables and figures. Table A. Summary table of the concentrations and incubation times for the different taxa from the refined staining experiment. **Fig A.** Probability of larval survival for *Acropora spathulata* exposed to two stains at different concentration levels and incubation times. Colors indicate the strength of the larval stain at each stage (larval stage and settled larvae; inset key indicates none, light, medium, or strong staining), error bars = standard error. Data underlying this Figure can be found at https://doi.org/10.25919/4rry-xg84. **Fig B.** Probability of larval survival and larval settlement for *Acropora spathulata* exposed to four stains (neutral red, Nile blue, alizarin red, and calcein blue) at different concentration levels after 12 hours of incubation and control (unstained) larvae. Colors indicate the strength of the larval stain (see Fig 2 for legend). Pairwise differences indicate significant differences from control (^ns^ = no significant difference, * = *p* < 0.05, ** *p* < = 0.01, *** = *p* < 0.001). Data underlying this Figure can be found at https://doi.org/10.25919/4rry-xg84. **Fig C.** Example of a settlement tile with newly settled *P*. *daedalea* 8 days after spawning following a mixed staining treatment of 50% neutral red stain, 50% Nile blue stain under a light microscope. Red scale bar = 1 mm. **Fig D.** Probability of larval settlement for four species of coral exposed to neutral red and Nile blue stains at different concentration levels and incubation times. Data underlying this Figure can be found at https://doi.org/10.25919/4rry-xg84. **Fig E.** Procedural approaches to stain larvae at laboratory and field scales. (a) Staining coral larvae in small separators that are nesting in varying concentrations of neutral red and Nile blue solutions in 6-well cell culture plate wells for easy removal at different times and rinsing following removal. (b) Mixing of Nile blue staining in seawater into which (c) larvae are retained in the stain within large separators for easy removal and rinsing prior to deployment.
(DOCX)
