# Supplementary material for: Coloring coral larvae allows tracking of local dispersal and settlement
Source: PLoS Biol. 2022 Dec 6;20(12):e3001907. doi: 10.1371/journal.pbio.3001907 (PMC9725161; doi:10.1371/journal.pbio.3001907)
Supplement: S1 Text — Table A. Summary table of the concentrations and incubation times for the different taxa from the refined staining experiment. Fig A. Probability of larval survival for Acropora spathulata exposed to two stains at different concentration levels and incubation times. Colors indicate the strength of the larval stain at each stage (larval stage and settled larvae; inset key indicates none, light, medium, or strong staining), error bars = standard error. Data underlying this Figure can be found at https://doi.org/10.25919/4rry-xg84. Fig B. Probability of larval survival and larval settlement for Acropora spathulata exposed to four stains (neutral red, Nile blue, alizarin red, and calcein blue) at different concentration levels after 12 hours of incubation and control (unstained) larvae. Colors indicate the strength of the larval stain (see Fig 2 for legend). Pairwise differences indicate significant differences from control (ns = no significant difference, * = p < 0.05, ** p < = 0.01, *** = p < 0.001). Data underlying this Figure can be found at https://doi.org/10.25919/4rry-xg84. Fig C. Example of a settlement tile with newly settled P. daedalea 8 days after spawning following a mixed staining treatment of 50% neutral red stain, 50% Nile blue stain under a light microscope. Red scale bar = 1 mm. Fig D. Probability of larval settlement for four species of coral exposed to neutral red and Nile blue stains at different concentration levels and incubation times. Data underlying this Figure can be found at https://doi.org/10.25919/4rry-xg84. Fig E. Procedural approaches to stain larvae at laboratory and field scales. (a) Staining coral larvae in small separators that are nesting in varying concentrations of neutral red and Nile blue solutions in 6-well cell culture plate wells for easy removal at different times and rinsing following removal. (b) Mixing of Nile blue staining in seawater into which (c) larvae are retained in the stain within large separators for easy removal [file pbio.3001907.s001.docx]

# **Coloring coral larvae allows tracking of local dispersal and settlement**

Christopher Doropoulos ^1, ¶, *^, George Roff ^1, ¶, *^

^1^ CSIRO Oceans & Atmosphere, St Lucia, Australia

^¶^ These authors contributed equally

* [christopher.doropoulos@csiro.au](mailto:christopher.doropoulos@csiro.au) (CD); [*george.roff@csiro.au](mailto:*george.roff@csiro.au) (GR)

# **Supplementary Table and Figures**

**Table A** Summary table of the concentrations and incubation times for the different taxa from the refined staining experiment.

| Taxa | Stain | Concentration (mg l^-1^) | Incubation time (minutes) | Larvae (*n*) | Replicates |
| --- | --- | --- | --- | --- | --- |
| *A. anthocercis* | Control | 0 | 30 | 10 | 3 |
|  |  | 0 | 120 | 10 | 3 |
|  | Neutral red | 1 | 15 | 10 | 3 |
|  |  | 10 | 10 | 10 | 3 |
|  |  | 10 | 30 | 10 | 3 |
|  |  | 100 | 5 | 10 | 3 |
|  |  | 100 | 10 | 10 | 3 |
|  | Nile blue | 10 | 60 | 10 | 3 |
|  |  | 100 | 60 | 10 | 3 |
|  |  | 500 | 60 | 10 | 3 |
|  |  | 500 | 120 | 20 | 3 |
|  |  | 1000 | 120 | 20 | 3 |
| *C. aspera* | Control | 0 | 105 | 20 | 3 |
|  | Neutral red | 10 | 20 | 20 | 3 |
|  |  | 100 | 10 | 20 | 3 |
|  | Nile blue | 500 | 105 | 20 | 3 |
|  |  | 1000 | 105 | 20 | 3 |
| *D. favus* | Control | 0 | 120 | 20 | 3 |
|  | Neutral red | 10 | 30 | 20 | 3 |
|  |  | 100 | 10 | 20 | 3 |
|  | Nile blue | 500 | 120 | 20 | 3 |
|  |  | 1000 | 120 | 20 | 3 |
| *P. sinensis* | Control | 0 | 105 | 20 | 3 |
|  | Neutral red | 10 | 20 | 20 | 3 |
|  |  | 100 | 10 | 20 | 3 |
|  | Nile blue | 500 | 105 | 20 | 3 |
|  |  | 1000 | 105 | 20 | 3 |


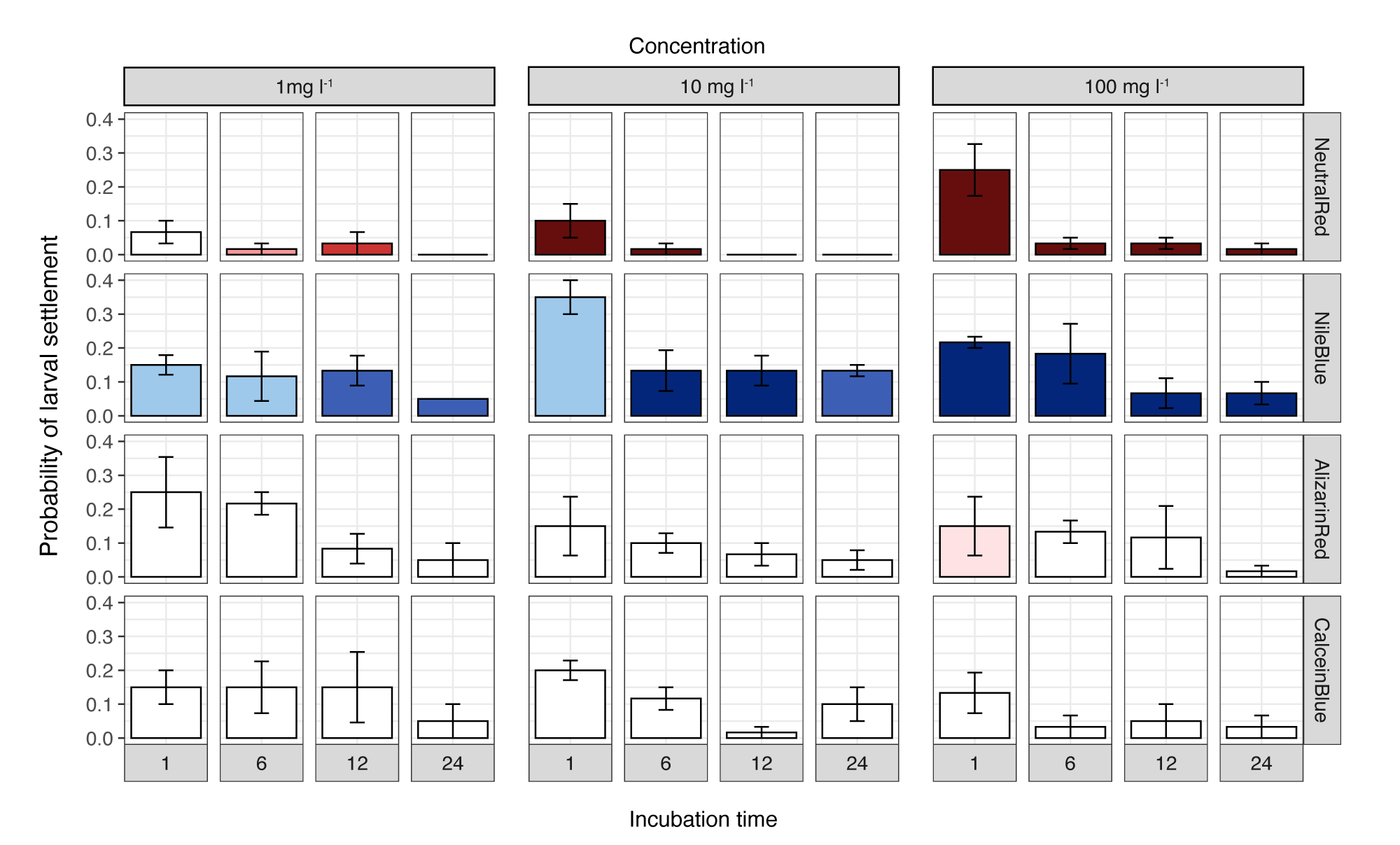


**Fig A** Probability of larval survival for *Acropora spathulata* exposed to two stains at different concentration levels and incubation times. Colors indicate the strength of the larval stain at each stage (larval stage and settled larvae; inset key indicates none, light, medium or strong staining), error bars = standard error. Data underlying this Figure can be found at <https://doi.org/10.25919/4rry-xg84>


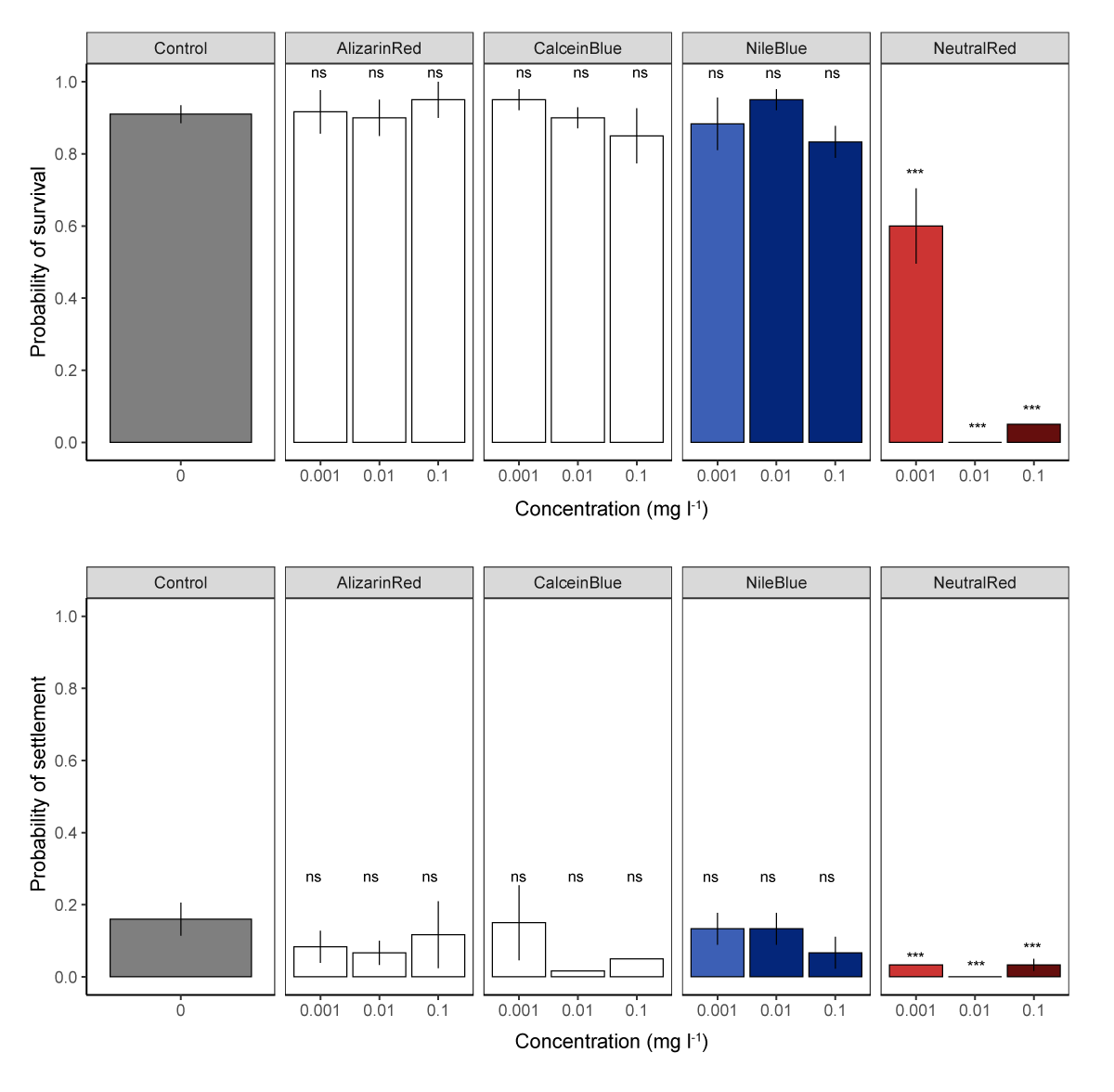
**Fig B** Probability of larval survival and larval settlement for *Acropora spathulata* exposed to four stains (neutral red, Nile blue, alizarin red, calcein blue) at different concentration levels after 12 hours of incubation and control (unstained) larvae. Colors indicate the strength of the larval stain (see Fig 2 for legend). Pairwise differences indicate significant differences from control (^ns^ = no significant difference, * = p < 0.05, ** p < = 0.01, *** = p <0.001). Data underlying this Figure can be found at <https://doi.org/10.25919/4rry-xg84>


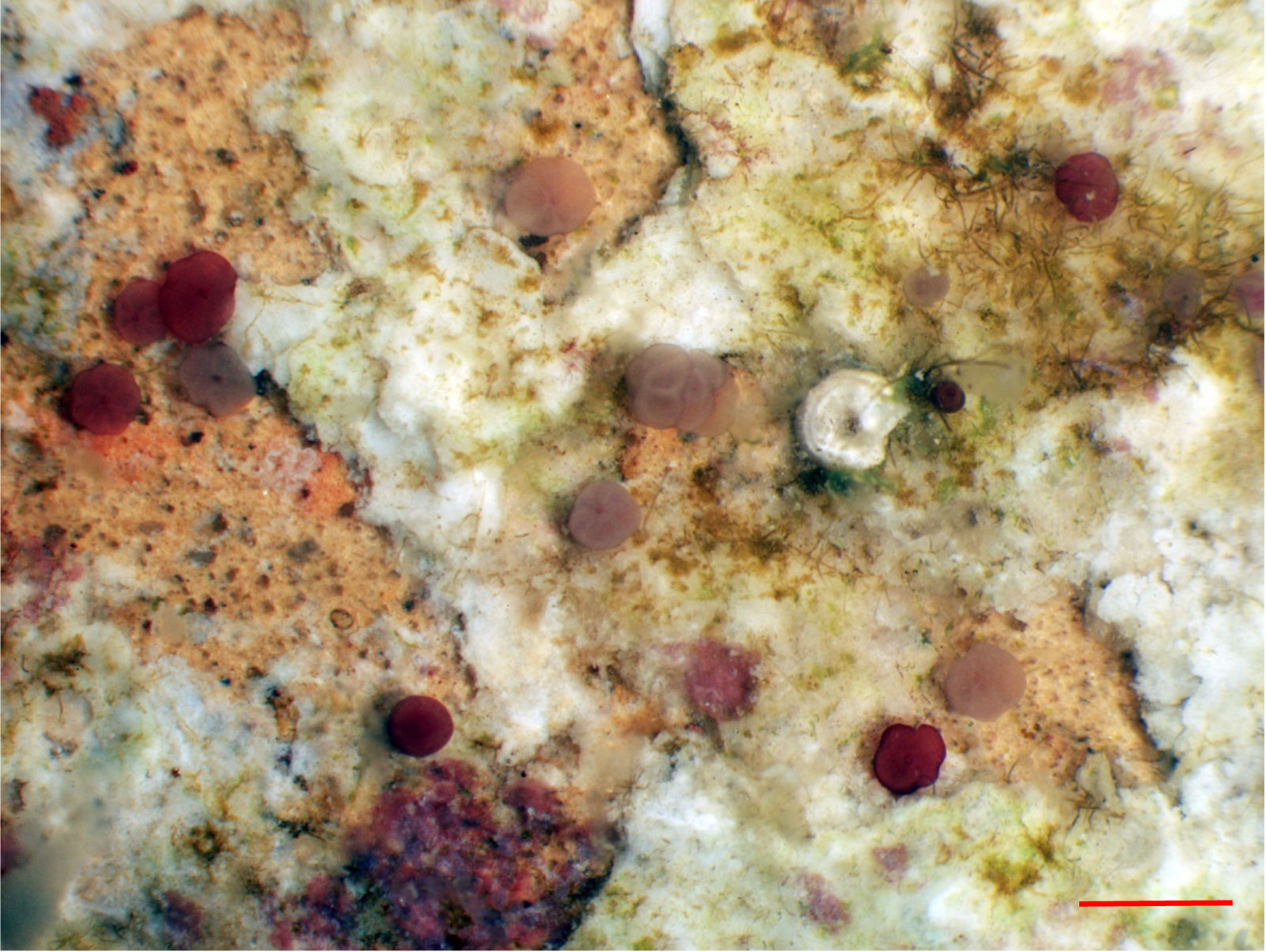


**Fig C** Example of a settlement tile with newly settled *Platygyra daedalea* 8 days after spawning following a mixed staining treatment of 50% neutral red stain, 50% Nile blue stain under a light microscope. Red scale bar = 1 mm.

**
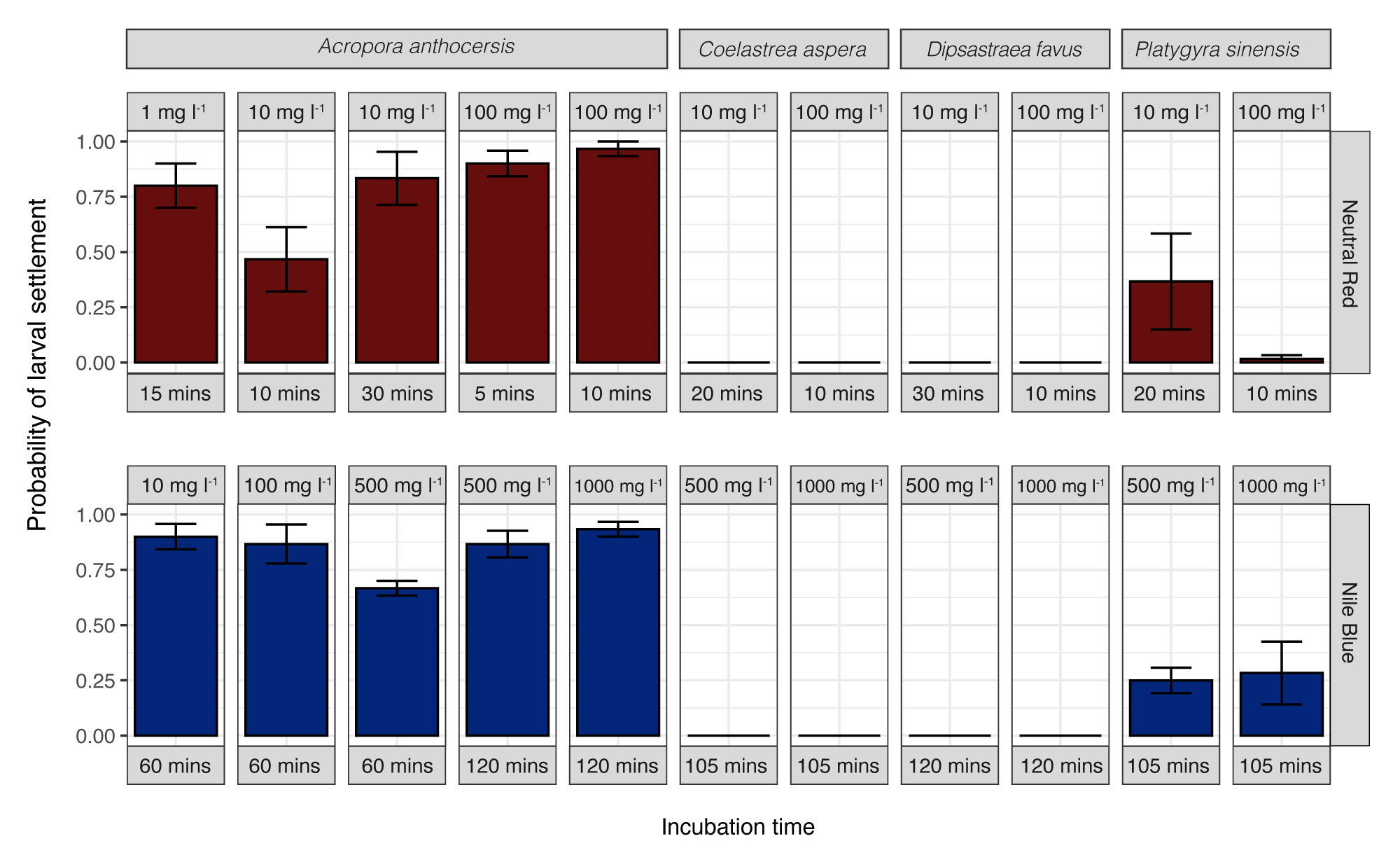
Fig D** Probability of larval settlement for four species of coral exposed to neutral red and Nile blue stains at different concentration levels and incubation times. Data underlying this Figure can be found at <https://doi.org/10.25919/4rry-xg84>


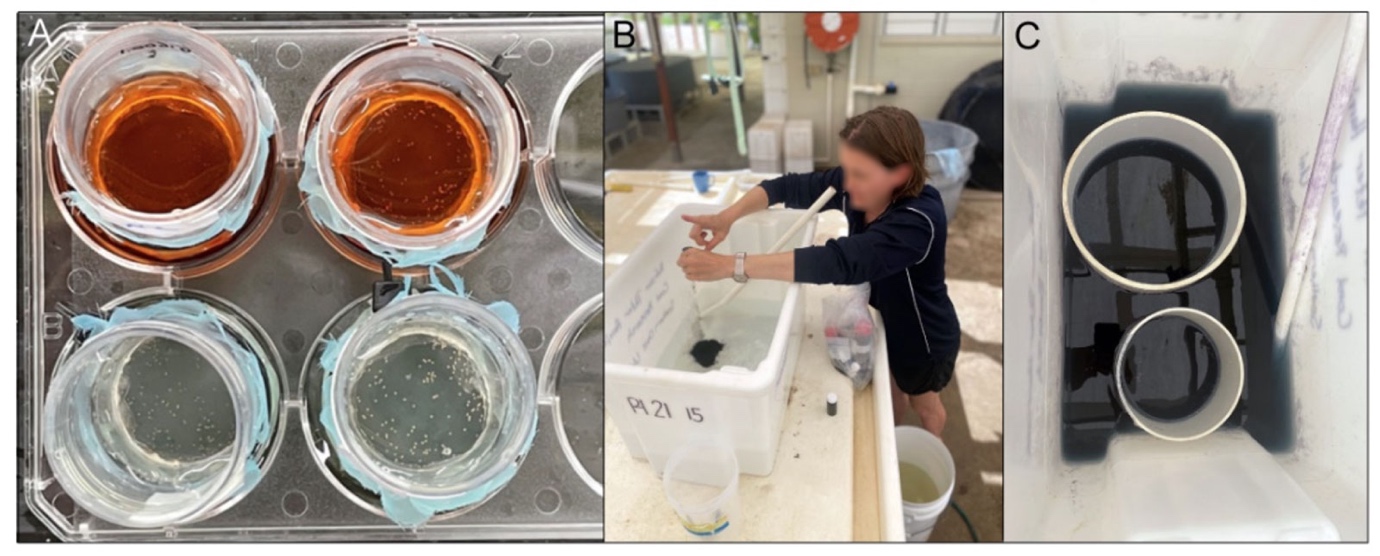


**Fig E** Procedural approaches to stain larvae at laboratory and field scales. a) Staining coral larvae in small separators that are nesting in varying concentrations of neutral red and Nile blue solutions in 6-well cell-culture plate wells for easy removal at different times and rinsing following removal. b) Mixing of Nile blue staining in seawater into which c) larvae are retained in the stain within large separators for easy removal and rinsing prior to deployment. Images supplied by authors.
